# HDAC1 Inactivation Induces Mitotic Defect and Caspase-Independent Autophagic Cell Death in Liver Cancer

**DOI:** 10.1371/journal.pone.0034265

**Published:** 2012-04-04

**Authors:** Hong Jian Xie, Ji Heon Noh, Jeong Kyu Kim, Kwang Hwa Jung, Jung Woo Eun, Hyun Jin Bae, Min Gyu Kim, Young Gyoon Chang, Jung Young Lee, Hanna Park, Suk Woo Nam

**Affiliations:** 1 Department of Pathology, College of Medicine, The Catholic University of Korea, Seoul, Korea; 2 Functional RNomics Research Center, College of Medicine, The Catholic University of Korea, Seoul, Korea; University of Illinois at Chicago, United States of America

## Abstract

Histone deacetylases (HDACs) are known to play a central role in the regulation of several cellular properties interlinked with the development and progression of cancer. Recently, HDAC1 has been reported to be overexpressed in hepatocellular carcinoma (HCC), but its biological roles in hepatocarcinogenesis remain to be elucidated. In this study, we demonstrated overexpression of HDAC1 in a subset of human HCCs and liver cancer cell lines. HDAC1 inactivation resulted in regression of tumor cell growth and activation of caspase-independent autophagic cell death, via LC3B-II activation pathway in Hep3B cells. In cell cycle regulation, HDAC1 inactivation selectively induced both p21^WAF1/Cip1^ and p27^Kip1^ expressions, and simultaneously suppressed the expression of cyclin D1 and CDK2. Consequently, HDAC1 inactivation led to the hypophosphorylation of pRb in G1/S transition, and thereby inactivated E2F/DP1 transcription activity. In addition, we demonstrated that HDAC1 suppresses p21^WAF1/Cip1^ transcriptional activity through Sp1-binding sites in the p21^WAF1/Cip1^ promoter. Furthermore, sustained suppression of HDAC1 attenuated *in vitro* colony formation and *in vivo* tumor growth in a mouse xenograft model. Taken together, we suggest the aberrant regulation of HDAC1 in HCC and its epigenetic regulation of gene transcription of autophagy and cell cycle components. Overexpression of HDAC1 may play a pivotal role through the systemic regulation of mitotic effectors in the development of HCC, providing a particularly relevant potential target in cancer therapy.

## Introduction

Hepatocellular carcinoma (HCC) is a primary malignancy of human liver and a major cause of morbidity and mortality. It is the seventh most common cancer worldwide, and the third leading cause of cancer-related deaths [1]. In the molecular mechanism, hepatocarcinogenesis is accepted as a multistep process characterized by the progressive accumulation and interplay of genetic alterations causing aberrant growth and malignant transformation of liver parenchymal cells, followed by vascular invasion and metastasis [2]. The global change signatures of the gene expression and signaling pathways, involved in HCC development, were investigated by many researchers. However, numerous genes which contribute to these alterations are still not characterized sufficiently.

Histone deacetylases (HDACs) are histone modifying enzyme families that regulate the expression and activity of numerous proteins involved in both cancer initiation and progression, by removing the acetyl groups, and thus allowing compact chromatin structure [3]. HDACs comprise a family of 18 genes, which are grouped into classes I-IV based on the homology to their respective yeast orthologues [4]. HDAC1, as a class I member sharing a high sequence homology with yeast Rpd3, is a global gene regulator and transcriptional co-repressor with histone deacetylase activity [5]. Aberrant expression of HDAC1 appears common in cancers of the gastrointestinal system, and is associated with dedifferentiation, enhanced proliferation, invasion, advanced disease and poor prognosis [4]. HCC patients with high expression of HDAC1 showed higher incidence of cancer cell invasion into the portal vein, poorer histological differentiation, more advanced tumor-node-metastasis (TNM) stage and low survival rate [6]. It was also found that highly expression of HDAC1 in cancer cells is correlated with chemotherapy resistance and poor prognosis in a series of carcinomas [7], . Silence of HDAC1 by small interference RNA (siRNA) or specific inhibitor MS-275 in cancer cells can either arrest at the G1 phase of the cell cycle or at the G2/M transition, resulting in the loss of mitotic cells, cell growth inhibition, and increase in the percentage of apoptotic cells [10], [11], [12]. In addition, HDAC1 knockdown affected cell motility and invasion by regulating E-cadherin expression [13], [14], and was also shown to induce autophagy in Hela cells [15], and cellular senescence in human fibroblast cells and prostate cancer cells [16]. Although these molecular functions of HDAC1 were well documented in numerous previous results, the role of HDAC1 in hepatocarcinogenesis has not been elucidated.

In the present study, in order to investigate the biological roles of HDAC1 that confer oncogenic potential in human HCC, we assessed the aberrant regulation of HDAC1 in a subset of human HCC tissues and examined the regulatory mechanisms of HDAC1 in apoptosis, autophagy and cell cycle of HCC cells. In addition, *in vitro* and *in vivo* experimental tumorigenic potential of HDAC1 were explored using stable HDAC1 knockdown cell lines.

## Results

### HDAC1 suppression causes mitotic defects in HCC cells

**Figure 1:**
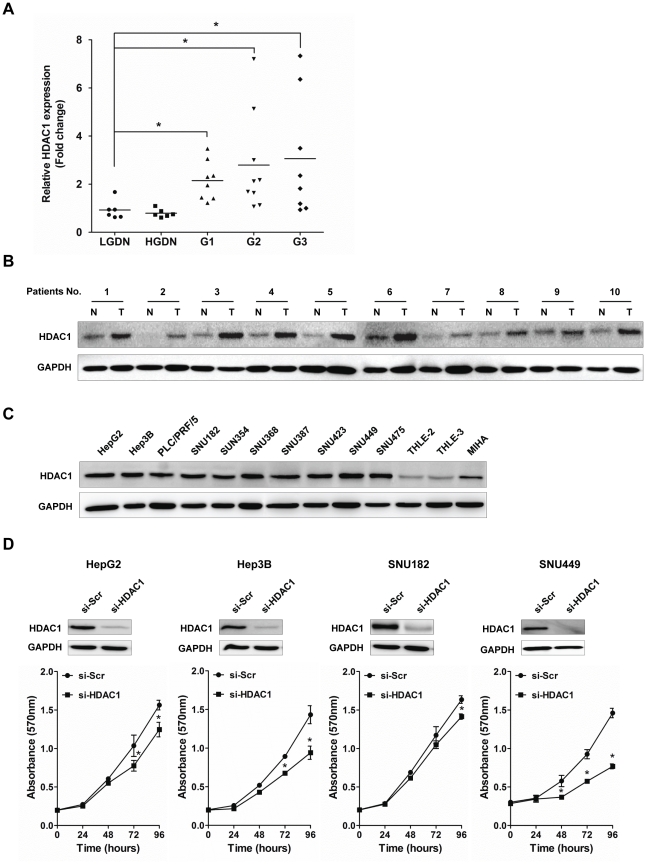
Aberrant expression of HDAC1 in human HCCs and liver cancer cell lines. (A) HDAC1 mRNA expression shows gradual increase from pre-cancer to the overt HCCs (*p<0.05). LGDN, low-grade dysplastic nodule; HGDN, high-grade dysplastic nodule; G1–3, Edmondson grades 1–3. (B) Ten pairs of tumor (T) and their corresponding non-tumor (N) liver tissues were obtained from surgical resections. The protein level of HDAC1 was assessed by Western blot analysis. (C) The endogenous levels of HDAC1 in ten liver cancer and three normal cell lines. An antibody against GAPDH served as loading control. (D) Targeted-disruption of HDAC1 causes the growth retardation of HCC cell lines. Cell viability was determined by MTT assay in the indicated cell lines transfected with either scramble (si-Scr) or HDAC1 siRNA (si-HDAC1). Data were expressed as mean ± SD (*p<0.01). All measurements were performed in triplicate, and each experiment was repeated at least two times.

We previously reported large-scale transcriptomic changes from preneoplastic lesion to overt human HCCs [17]. From primary microarray data, we recapitulated the expression of HDAC1 in a multi-step histopathological process, from low-grade dysplastic nodules (LGDNs) and high-grade dysplastic nodules (HGDNs) to primary HCC (Edmondson grades 1–3). As shown in Figure 1A, the relevant expression of HDAC1 was gradually increased from non-tumor to overt cancer. To confirm the overexpression of HDAC1 in HCC, we performed immunoblot analysis of HDAC1 in a subset of human HCCs. As shown in Figure 1B, HDAC1 appeared to be highly overexpressed in all selected HCC tissues compared to the corresponding non-cancerous tissues. Expression of HDAC1 was also analyzed in ten different HCC cell lines (HepG2, Hep3B, PLC/PRF/5, SNU182, SNU354, SNU368, SNU387, SNU423, SNU449 and SNU475) and compared with three selective immortalized normal liver hepatocyte cell lines (THLE-2, THLE-3 and MIHA). As shown in Figure 1C, endogenous expression of HDAC1 in all HCC cell lines exhibited relatively higher than that of normal liver hepatocyte cell lines.

Next, to explain the biological consequences of aberrant expression of HDAC1 in hepatocarcinogenesis, HDAC1 expression was abrogated by the RNA-interference mediated gene knock-down in four different HCC cell lines; HepG2, Hep3B, SNU182 and SNU449 cells. As shown in Figure 1D, HDAC1 depletion resulted in the significant reduction of tumor cell growth (HepG2, Hep3B and SNU449). This anti-growth effect could be partially explained by the disruption of cell growth regulation, such as cell cycle arrest, cellular senescence or apoptosis. Thus, we next explored the effects of HDAC1 suppression on the cell cycle regulation and cellular apoptosis.

### Aberrant expression of HDAC1 mediated proliferation of liver cancer cells by deregulating expression of G1/S cell cycle proteins

**Figure 2:**
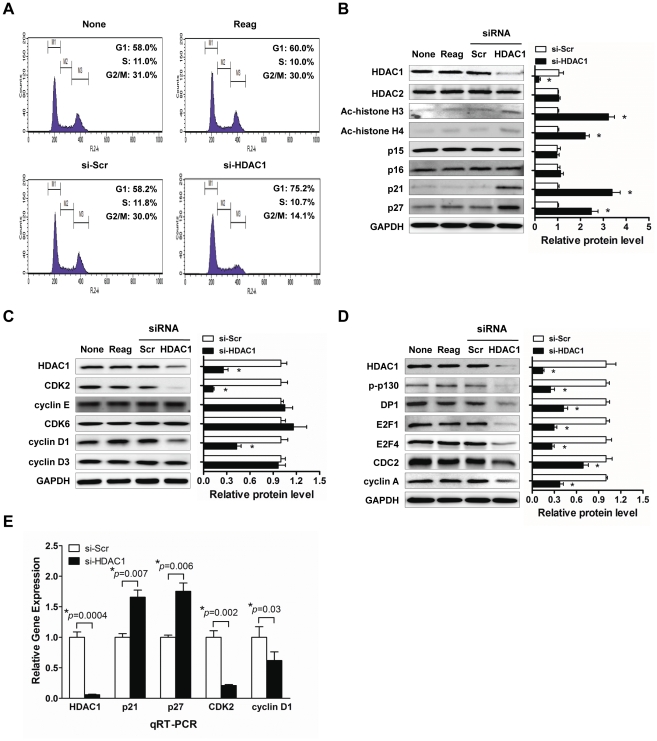
HDAC1 controls the expressions of regulatory components in G1/S cell cycle transition. Hep3B cells were transfected with control (None), lipofectamine only (Reag), 100 nmol/L scrambled siRNA (si-Scr) or 100 nmol/L of HDAC1-specific siRNA (si-HDAC1). (A) The flow cytometric analysis was conducted with propidium iodide (PI) staining on suppression of HDAC1 in Hep3B cells. Two independent experiments with the same results were performed. (B) To ascertain the suppression of HDAC1 activity, protein expressions of acetyl-histone H3 and H4 were determined by immunoblotting. Protein expressions of negative regulators in G1/S cell cycle transition were also assessed in HDAC1-depleted Hep3B cells. (C) Suppression of CDKs and cyclins in G1/S transition by HDAC1 depletion. (D) Effects of HDAC1 depletion on pRb and E2F/DP1 target gene expressions. (E) Validation of the mRNA expression of HDAC1 regulated genes by quantitative real-time PCR analysis. The relative expression level of each gene was normalized to GAPDH mRNA in the same sample. Protein expression level was quantified by ImageJ and normalized to GAPDH. The values are given as fold-change relative to the si-Scr tansfectants. The relative proteins level is shown as bar graphs in right side of each immunoblot (*p<0.05). All experiments were repeated three times with the same results. A typical result of three performed experiments is shown.

The fact that the suppression of HDAC1 caused regression of liver cancer cell growth implies that HDAC1 is involved in the regulation of the cell cycle progression. Thus, we performed cell cycle analysis of PI-stained cells in HDAC1 siRNA transfectants using flow cytometry. HDAC1 depletion led to an increase in G1 phase by 17.0% with a concomitant decrease in S phase and G2/M phase by 1.1% and 15.9%, respectively compared to the control (si-Scr) at 48 h after transfection (Figure 2A). The fact that the suppression of HDAC1 caused cell cycle arrest in G1 phase implies that HDAC1 can modulate activities of cell cycle regulating components. Therefore, we examined the effects of HDAC1 depletion on the regulatory proteins of G1/S cell cycle transition. In G1/S transition, it has been well established that negative cell cycle regulators, such as p15^INK4B^, p16^INK4A^, p18^INK4C^, p19^INK4D^, p21^WAF1/Cip1^ and p27^Kip1^, are the key modulators that suppress cyclin D1/CDK4 and 6, or cyclin E/CDK2 complexes. When these cell cycle modulators were examined, HDAC1 depletion selectively caused the induction of p21^WAF1/Cip1^ and p27^Kip1^ among the negative regulators of cell cycle transition in Hep3B cells (Figure 2B). Notably HDAC1 inactivation by using HDAC1 siRNA did not affect endogenous expression of HDAC2, and it also caused accumulation of acetylated histone H3 and H4 implying HDAC1 depletion-specific induction of p21^WAF1/Cip1^ and p27^Kip1^ in Hep3B cells. In addition, HDAC1 depletion also elicited the concomitant suppression of CDK2 and cyclin D1 (Figure 2C). In general, the activated CDK/cyclin complex can cause hyperphosphorylation of pRb, which loses its tumor suppressor activity, and which allows for the increase of E2F/DP1 transcriptional activity. Thus, we next investigated whether dysregulation of CDK and cyclins by HDAC1 affects the E2F/DP1 transcriptional activity in Hep3B cells. Targeted-disruption of HDAC1 elicited hypophosphorylation of p130, a pRb isoform (Rb2). Consequently, some of the downstream target genes of E2F/DP1 transcription factor, such as E2F4, CDC2 and cyclin A, were significantly down-regulated (Figure 2D). Further, to validate these results and to confirm transcriptional levels of differentially expressed genes, we performed quantitative real-time PCR (qRT-PCR) for *HDAC1*, *CDKN1A* (p21^WAF1/Cip1^), *CDKN1B* (p27^Kip1^), *CDK2* and *CCND1* (cyclin D1) genes. As shown in Figure 2E, HDAC1-depleted Hep3B cells exhibited very low endogenous HDAC1 expression. We were also able to confirm that both *CDKN1A* (p21^WAF1/Cip1^) and *CDKN1B* (p27^Kip1^) were significantly up-regulated by HDAC1 inactivation. Inversely, *CDK2* and *CCND1* were appeared to be down-regulated by HDAC1 inactivation (Figure 2E). These results suggest that exclusive regulation of cell cycle proteins, such as p21^WAF1/Cip1^, p27^Kip1^, cyclin D1 and CDK2 by HDAC1 overexpression exerts a very potent mitogenic stimulation during liver cancer progression.

**Figure 3:**
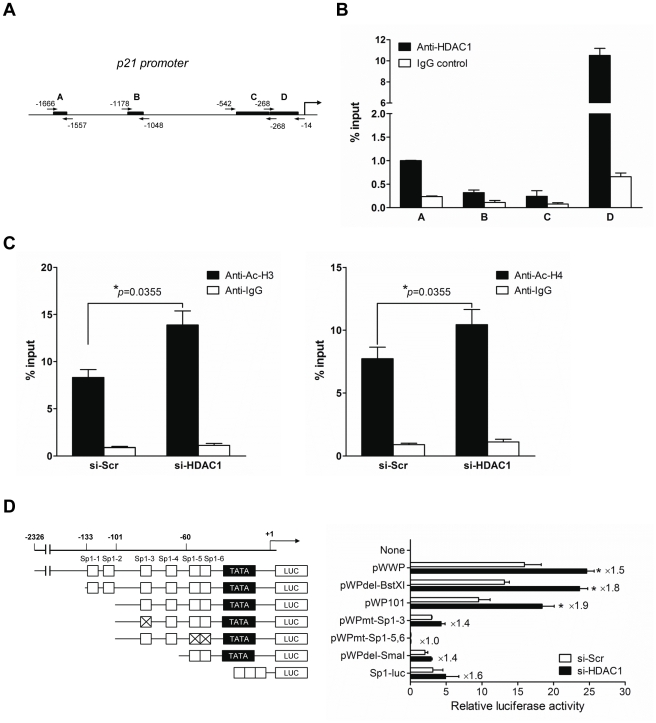
HDAC1 regulates p21^WAF1/Cip1^ transcription via Sp1-binding sites in the p21^WAF1/Cip1^ promoter. (A) A schematic of the p21^WAF1/Cip1^ promoter depicting the regions analyzed by ChIP-qPCR (black bars, A–D). (B) The association of HDAC1 in the p21^WAF1/Cip1^ promoter was assessed by the amplification of each region immunoprecipitated with HDAC1. The amount of DNA precipitated by either anti-HDAC1 or control IgG was expressed as percentage of the total input genomic DNA. The result of four independent experiments was shown as mean ± SD. (C) Acetylations of histone H3 and H4 associated with the proximal p21^WAF1/Cip1^ promoter was increased by inhibiting association of HDAC1. Hep3B cells were transiently transfected with control (si-Scr) or HDAC1 siRNA (si-HDAC1) for 48 h, and subjected to ChIP-qPCR analysis using acetyl-histone H3 (anti-Ac-H3) or H4 (anti-Ac-H4) antibody or control IgG. Precipitated genomic DNA was amplified for the proximal promoter of the p21^WAF1/Cip1^ locus (region D) by real-time PCR. The amount of precipitated DNA was expressed as percentage of the total input genomic DNA. The result of three independent experiments was shown as mean ± SD. (D) HDAC1 regulates p21^WAF1/Cip1^ transcription via Sp1-binding sites in the p21^WAF/Cip1^ promoter. The indicated constructs, pWWP, pWPdel-BstXI, pWP101, pWPmt-Sp1-3, pWPmt-Sp1-5,6, pWPdel-SmaI and Sp1-luc were transiently transfected into Hep3B cells, with 100 nmol/L scrambled siRNA (si-Scr) or 100 nmol/L HDAC1 specific siRNA (si-HDAC1), respectively. The promoter activity was measured, and fold induction by HDAC1 depletion was calculated. On the left, the scheme of each construct was shown. All the data were shown as the mean ± SD (n = 3) (*p<0.05).

Several studies have shown that HDAC inhibitors strongly activate the expression of p21^WAF1/Cip1^ through enhanced histone acetylation of the p21^WAF1/Cip1^ promoter including Sp1- binding site by releasing the repressor HDAC from its binding [18], [19]. Furthermore, there were some evidences that the endogenous expression of p21^WAF1/Cip1^ is regulated at the transcription level by the recruitment of HDAC1 to the p21^WAF1/Cip1^ promoter region [20], [21]. Our present results also suggest that the transcriptional suppression of p21^WAF1/Cip1^ through HDAC1 binding on its promoter region is predominant in liver cancer cells. To validate this implication, we performed chromatin immunoprecipitation assay with quantitative PCR (ChIP-qPCR) for indicated p21^WAF1/Cip1^ promoter regions which had been analyzed in our previous report (Figure 3A) [22]. We found that HDAC1 is highly associated with the proximal region of the p21^WAF1/Cip1^ promoter (region D in Figure 3B). This result was further validated by ChIP-qPCR analysis with the same promoter region of p21^WAF1/Cip1^ (region D), and which showed that the acetylation status of histone H3 and/or H4 is enhanced on the genomic locus by the abrogation of HDAC1 (Figure 3C).

As described above, there are enriched Sp1-biding sites in region D. So, we next investigated whether HDAC1 suppresses p21^WAF1/Cip1^ expression via Sp1-binding sites on the p21^WAF1/Cip1^promoter. To confirm this, we performed the reporter assay with reporter constructs described in materials and methods. As shown in Figure 3D, HDAC1-deficient Hep3B cells (si-HDAC1) exhibited increased luciferase activity in the presence of Sp1-binding sites, at least containing Sp1-3 to Sp1-6 (pWWP, pWPdel-BstXI and pWP101). However, p21^WAF1/Cip1^ transcription activity was not induced by mutant forms of Sp1-5,6 (pWPmt-Sp1-5,6) in si-HDAC1 cells. Interestingly, p21^WAF1/Cip1^ transcription was still induced by two mutant plasmids (i.e. pWPdel-SmaI and Sp1-luc) having two or three tandem repeats of the Sp1-binding site near the TATA box or transcription start site. This result indicates that the proximal region around the TATA box of p21^WAF1/Cip1^ promoter is expected to be an important site regulating p21^WAF1/Cip1^ transcription by HDAC1 in Hep3B cells.

### Targeted-inhibition of HDAC1 induces autophagic cell death of Hep3B cells

**Figure 4:**
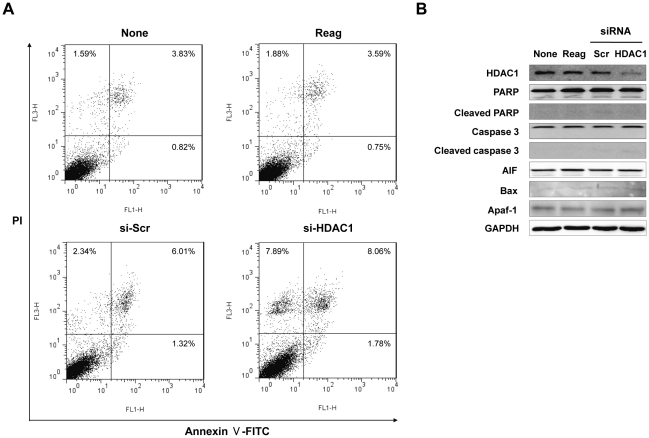
HDAC1 inactivation does not induce apoptotic cell death in Hep3B cells. (A) The flow cytometric analysis was conducted through Annexin V-FITC labeling and PI staining on suppression of HDAC1. Double staining with Annexin V-FITC and PI indicates that the amount of cells in the late stage of apoptosis was slightly increased (Upper right) in HDAC1-depleted Hep3B cells. Two independent experiments with the same results were performed. (B) Both PARP and caspase 3 cleavage were not detected by HDAC1 inhibition. All experiments were repeated three times with the same results.

Recent studies suggested that the treatment with histone deacetylase inhibitors induced not only apoptotic, but also autophagic cell death [23], [24], [25]. It was also found that knockdown of HDAC1 induces autophagic cell death in Hela cells [15]. Thus, we explored the effects of HDAC1 suppression on the activation of cell death in Hep3B cells. As shown in Figure 4A, flow cytometric analysis for measuring Annexin V stained cells showed no significant induction of apoptotic cells compared to control (si-Scr). Additionally, HDAC1 depletion did not affect the expressions of pro-apoptotic components, such as AIF, Bax and Apaf-1, nor did it cause caspase-3 and PARP cleavage (Figure 4B). These results indicated that overexpression of HDAC1 did not mainly affect the apoptotic signal in HCC.

**Figure 5:**
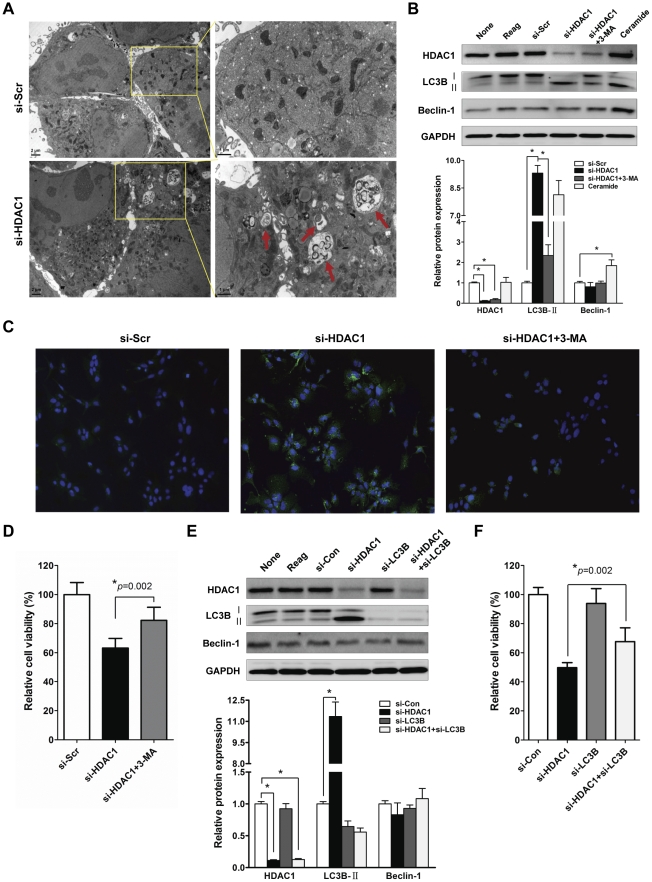
HDAC1 inactivation causes autophagic cell death in Hep3B cells. (A) Hep3B cells were transfected with HDAC1 siRNA (100 nmol/L) for 72 h, fixed and reviewed under the transmission electron microscope (TEM) (left two panels). With a higher magnification, autophagosomes containing electron dense material and degraded organelles were observed in HDAC1-deficient cells. In contrast, a lot of intact mitochondria were observed in Hep3B cells transfected by si-Scr (right two panels). Arrows indicate the presence of autophagosomes. (B) After knockdown of HDAC1, LC3 conversion (LC3B-I to LC3B-II) was markedly increased, whereas the level of Beclin 1 did not change. Parallel treatment with 5 mM 3-MA for 48 h inhibited LC3B-II activation induced by HDAC1 knockdown. Hep3B cells were also treated with 25 µM ceramide for 24 h as a positive control for the autophagy. The densitometry result of the immunoblots was shown as a bar graph (mean ± SD). Relative protein expressions of HDAC1, LC3B-II and Beclin 1 were normalized with the expression level of control (si-Scr) (*p<0.05). (C) In the immunofluorescence analysis, the cytosolic expression of LC3B was induced by HDAC1 knockdown, and which was reversed by the treatment with 3-MA in Hep3B cells. (D) MTT analysis shows that cell viability was regressed by HDAC1 knockdown in Hep3B cell. The number of viable cells was increased in the parallel treatment of 3-MA for 48 h (*p<0.05). (E) Co-transfection of siRNA targeting LC3B and/or HDAC1. The LC3B conversion was induced by si-HDAC1 in the absence of si-LC3B. The level of proteins were quantified by ImageJ and normalized to GAPDH. Bar graph shows the relative ratio to si-Con transfectant (*p<0.05). (F) MTT analysis shows that the cells viability was recovered apparently in the presence of si-LC3B in HDAC1 knockdown Hep3B cells (*p<0.05, si-HDAC1+si-LC3B vs si-HDAC1).

Next, to determine whether HDAC1 inactivation causes activation of autophagic cell death, we examined the microscopic structure of cells by transmission electron microscopy (TEM) in HDAC1-depleted Hep3B cells. As expected, approximately 40–45% of the HDAC1 siRNA-transfected cells developed autophagic vacuoles after 72 h of post-transfection (Figure 5A). At higher magnifications, most vacuoles contained electron dense material and degraded organelles. In contrast, control siRNA (si-Scr) transfected-cells were merely vacuolated, and fewer cells containing vacuoles in cytoplasm were observed (right two panels in Figure 5A). Microtubule-associated protein 1 light chain 3 (LC3) is a common marker for detecting autophagy. LC3 is post-translationally modified by removal of its C-terminus to produce LC3-I (∼18 kDa), with phosphatidylethanolamine lipidation giving rise to LC3-II (∼16 kDa). Enrichment of membrane-bound LC3-II in autophagosomes is a distinctive phenomenon of autophagic cell death and the amount of LC3-II correlates well with the number of autophagosomes. In our experiments, HDAC1 knockdown significantly increased the conversion of LC3B-I into LC3B-II as much as ceramide, a potent autophgy inducer, did in Hep3B cells (Figure 5B). In contrast, treatment with 3-methyladenine (3-MA; a specific inhibitor of autophagy) prevented LC3B-I to LC3B-II conversion by HDAC1 inactivation in Hep3B cells (lane 5 in Figure 5B). In addition, immunofluorescence staining for LC3B revealed that HDAC1 inactivation induced ring-shaped spots evenly distributed throughout cytoplasm, indicating an association between LC3 and autophagosomal membranes, and this association was significantly blocked by 3-MA treatment (Figure 5C). Consistent with this result, reduced cell viability caused by HDAC1 inactivation was effectively blocked by 3-MA treatment (Figure 5D). These results suggest that the HDAC1 knockdown activates autophagic cell death in HCC cells. Interestingly, we also found that HDAC1 inactivation did not affect Beclin-1 which participates during the early stages of autophagy, and that it promotes the nucleation of autophagic vesicles, and the recruitment of proteins from cytosol [26]. This implicated that HDAC1 inactivation induced Beclin-1 independent autophay in Hep3B cells. To verify that HDAC1 inactivation mediates LC3B-dependent autophagic cell death, LC3B was silenced in Hep3B cells. As shown in Figure 5E, LC3B-II conversion induced by HDAC1 inactivation was completely blocked by LC3B knockdown in Hep3B cells. Further, the decreased cell viability by HDAC1 inactivation was significantly restored by the co-transfection of LC3B siRNA in Hep3B cells (Figure 5F). Collectively, these results suggest that HDAC1 inactivation contributes caspase-independent cell death through LC3-dependent autophagy in Hep3B cells.

### Sustained-suppression of HDAC1 attenuates tumorigenic potential of Hep3B cells both *in vitro* and *in vivo*

**Figure 6:**
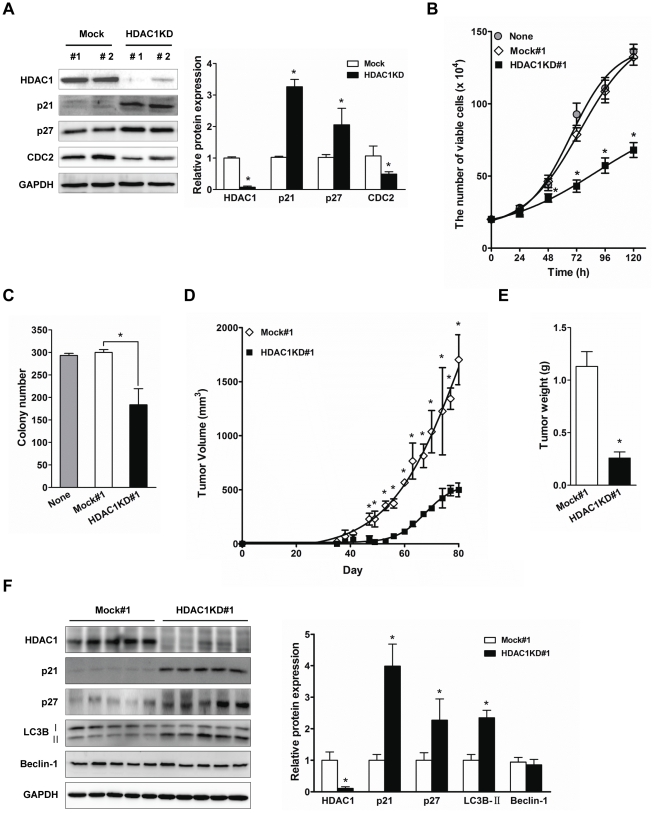
Sustained suppression of HDAC1 abrogates the tumorigenic potential of Hep3B cells. (A) The confirmation of HDAC1 suppression by detecting its specific target genes in cell cycle regulation. A typical result of three independent experiments was shown. The protein expressions were quantified and normalized to GAPDH, and were shown as relative ratio to Mock. (B) Cell growth rate of Hep3B cells stably expressing a scrambled shRNA (Mock#1) or HDAC1 shRNA (HDAC1KD#1) was determined by counting assay at each indicated time point. Data were presented as mean ± SD for three experiments. (C) *In vitro* colony formation assay. Cell clones expressing scrambled shRNA (Mock#1) or HDAC1 shRNA (HDAC1 KD#1) were maintained in 6-well plates. The graph bar indicates the number of colonies. Data are presented as mean ± SD from three independent experiments (*p<0.05, HDAC1KD#1 vs Mock#1). (D) Overall tumor growth of Hep3B cell xenografts. (E) Mice were sacrificed at eighty days after injection, and the tumor weight was measured. Data were presented as mean ± SD (*p<0.05). (F) At the moment of sacrifice, xenograft tumors were excised, and total protein extracted from five randomly selected mice of each group was subjected to Western blot analysis using the indicated antibodies. The protein expressions were normalized to those of GAPDH, and the relative expression level of each protein was depicted as bar graph (*p<0.05).

Our results showed that the transient disruption of HDAC1 caused *in vitro* autophagic cell death and growth arrest in Hep3B cells. Thus, to investigate whether the stable suppression of HDAC1 leads to suppression of hepatocarcinogenesis, we established stable HDAC1 knockdown cell lines (HDAC1KD#1 and HDAC1KD#2), and confirmed the inactivation of HDAC1 by detecting p21^WAF1/Cip1^ and p27^Kip1^ induction and CDC2 reduction in established cell lines (Figure 6A). We then assessed the growth rate of the established cell lines. As shown in Figure 6B, HDAC1KD#1 cells exhibited reduced growth rate, as compared to either Mock-transfectant (Mock#1) or Hep3B parental cells (None). Based on this result, we next performed the colony forming assay as described in materials and methods. The clonal cell growth was significantly attenuated by the sustained suppression of HDAC1 in HDAC1KD#1 cells, as compared to the control (Mock#1) (Figure 6C, p<0.05). Finally, to demonstrate that HDAC1 overexpression contributes to hepatocarcinogenesis *in vivo*, we subcutaneously injected established cell lines (i.e. HDAC1KD#1 or Mock#1) into athymic nude mice. The overall tumor growth rate and volume were significantly reduced in HDAC1 deficient cell line (HDAC1KD#1) compared to the control (Mock#1) (Figure 6D, p<0.05). At 37 days post-inoculation, tumor mass was detectable in the Mock group. In contrast, in the mice that were injected with HDAC1KD#1, tumor mass was detectable only at 48 days post-inoculation. The average tumor volume at sacrifice was much smaller in the group injected with HDAC1KD#1 than Mock#1 cells (Figure 6E, p<0.05). In addition, immunoblot analysis revealed that the expressions of p21^WAF1/Cip1^, p27^Kip1^ and LC3B-II were significantly induced in HDAC1 deficient xenograft tissues (Figure 6F, p<0.05). These results imply that the suppression of HDAC1 contributes to the inhibition of hepatocarcinogenesis *in vivo*.

## Discussion

Histone deacetylases (HDACs) represent a family of enzymes that cooperate with histone acetyltransferase to modulate chromatin structure and transcriptional activity via changes in the acetylation status of nucleosomal histones [27]. Accumulating evidences have suggested that HDACs regulate both the expression and activity of numerous proteins involved in both cancer initiation and progression [27], [28]. Recently, several studies have shown that certain HDAC families are aberrantly expressed in tumors and have redundant function in cancer development [4], [29]. Consistently, increasing evidence suggested the aberrant expression of HDAC1 in neoplastic diseases, and demonstrated that HDAC1 depletion causes growth arrest and apoptosis of certain human cancer cells [10], [27], [29]. However, the role of HDAC1 in hepatocarcinogenesis has not been elucidated yet. In this study, we have suggested that the abrogation of aberrant expression of HDAC1 activated the caspase-independent autophagic cell death and arrested the G1/S cell cycle transition in human Hep3B cells, and consequently suppressed the tumor cell growth in the xenograft animal model.

In a recent study of gastric cancer, HDAC1 expression was reported to be overexpressed and had prognostic value for gastric cancer [8]. Induction of HDAC1, 2, 3 expression levels also implicated significantly reduced patient survival in colorectal cancer [30]. The contribution of the HDAC1 expression to the malignant transformation of normal liver was also studied in a transgenic mouse model [31]. Furthermore, a recent report suggested that both HDAC1 and HDAC2 cooperatively regulated the proliferation of mouse embryonic fibroblast (MEF) and had an essential role for the hematopoietic differentiation [32]. In hepatocellular carcinoma, high HDAC1 expression was associated with cancer cell invasion into the portal vein, poor histological differentiation and low patient survival [6]. This was also confirmed by our previously published comprehensive mRNA-based expression microarray data [17]. Of these outlier genes associated with multi-step hepatocarcinogenesis, we recapitulated HDAC1 expression and showed highly overexpression in overt HCC. Immunoblot analysis of HDAC1 in a subset of human HCC tissues and liver cancer cell lines demonstrated overexpression of HDAC1 in HCCs, and targeted-disruptions of HDAC1 caused growth retardation in various HCC cell lines (Figure 1). The accumulating evidences suggest that HDAC1 overexpression appears especially common in cancers of the gastrointestinal system and is associated with dedifferentiation, enhanced proliferation, invasion, advanced disease and poor prognosis. However, no attempt has been made to explain the underlying mechanisms responsible for the oncogenic potential of HDAC1 in HCC. We therefore assessed effects of HDAC1 inactivation on the regulatory proteins in the cell growth and death mechanism.

It has been well reported that HDAC-mediated repression of genes can cause uncontrolled cell growth, as HDACs repress the transcription of cyclin-dependent kinase inhibitors (CDKIs), allowing continued proliferation [27]. In osteosarcoma and breast cancer cells, it was reported that the knockdown of HDAC1 resulted in the cell cycle arrest either at the G1 or G2/M phase transition, and increased the percentage of apoptotic cells [10]. Our results indicated that disruption of HDAC1 induced p21^WAF1/Cip1^ and p27^Kip1^ expressions thereby implying the inhibition of G1/S transition of cell cycle (Figure 2B). In addition, HDAC1 knockdown suppressed the expression of cyclin D1 and CDK2 (Figure 2C). Although it is not clear whether HDAC1 suppresses the cyclin D1 and CDK2 directly or through the induction of p21^WAF1/Cip1^, it is obvious that this synergistic negative regulation of G1/S transition suggests a potent role of HDAC1 in cell cycle regulation. Orderly progression through the cell cycle checkpoints involves coordinated activation of the CDKs, in the presence of an associated CDK-activating kinase, by phosphorylating the target substrates including members of the “pocket protein” family. One of these, the product of the retinoblastoma susceptibility gene (the pRb protein) is phosphorylated sequentially by the kinase activities of CDK4/cyclin D1 and CDK2/cyclin E complexes. Our results also demonstrated that the resulting suppression of CDK/cyclin complex augmented the hypophosphorylation status of p130, an isoform of pRb (Rb2), and suppressed transcriptional activation of E2F/DP1 target genes (Figure 2D).

HDACs were known to function by interacting with the tumor suppressor genes such as p53, pRb and BRCA1 [33], [34], [35]. For example, HDAC1 is necessary for the repression of E2F target genes by the interaction with pRb [33]. Alternatively, the deacetylation of non-histone proteins, such as p53, may also play a role in controlling the cell cycle dynamics. Unlike this suppressor role of HDAC1 on pRb and its interaction with E2F/DP transcription, our data suggests that HDAC1 acts as a potent modulator, suppressing the expression level of CDK inhibitor and augmenting cyclins and CDKs of the cell cycle circuit, especially in the G1/S transition in HCC. In addition, our results also demonstrated that the suppression of HDAC1 induces p21^WAF1/Cip1^via Sp1-binding site of its promoter region in a p53-independent manner (Hep3B is a p53-null cell line). Although it is not clear that HDAC1 directly suppresses p21^WAF1/Cip1^ through Sp1-binding site or does so indirectly by forming a complex with other molecules, it is obvious that HDAC1 suppression causes its release from the Sp1-binding site around the TATA box (Sp1-5,6), which leads to the enrichment of acetylated histones at the proximal region of the p21^WAF1/Cip1^ promoter (Figure 3).

Interestingly, unlike previous studies demonstrating the increase of apoptosis by HDAC1 inhibition, our results indicated that HDAC1 inactivation did not induce apoptosis (Figure 4) in liver cancer. Recently, HDAC1 inactivation was shown to induce autophagy in Hela cells [15]. With the TEM analysis for investigating microscopic structure of the cells with HDAC1 depletion, we could expect that HDAC1 suppresses the formation of autophagosome, which leads to the inhibition of autophagic cell death in HCC cells (Figure 5A). Further, we found that a part of autophagic cell death was modulated by HDAC1 through the activation of LC3 conversion (LC3-I to LC3-II) (Figures 5B–F). Given the inherent resistance to apoptosis that characterizes cancer, the targeting of alternative pathways is an attractive strategy to improve anti-tumor therapy. Our data showed that HDAC1 suppresses both cell cycle arrest and caspase-independent autophagic cell death in liver cancer cells. Therefore, these suppressive roles of HDAC1 may confer the effective oncogenic potential upon the cells, which increases the transforming activity during hepatocarcinogenesis.

In conclusion, we have shown the aberrant expression of HDAC1 in human HCC. We have also demonstrated that the targeted disruption of HDAC1 led to the strong anti-proliferative effect and induced autophagic cell death in HCC. Detailed analyses of the molecular mechanisms governing gene regulation by HDAC1 will illustrate how this protein influences proliferation and autophagic cells death. From those interpretations, we derived a conclusion that the aberrant actions of HDAC1 disturbed homeostasis via dysregulation of autophagy and cell cycle signaling in hepatocytes. Taken together, our present results underlined the tremendous potential of HDAC1 for enhancing our understanding of the intricate mechanisms of cell death as well as mitogenic stimulation of liver cells in the development of HCC, thereby suggesting HDAC1 as a novel target for therapeutic intervention.

## Materials and Methods

### Ethics Statement

Total ten hepatocellular carcinoma tissues with their corresponding normal were obtained from Yonsei University, School of Medicine, Seoul, Korea. Informed consent form was provided according to the Declaration of Helsinki. This study was approved by Institutional Review of Board of the Songeui Campus, College of Medicine, The Catholic University of Korea (IRB approval number; CUMC10U036). For the animal study, all animal experiments were performed with the approval of the Institutional Animal Care and Use Committee (IACUC) of Department of Laboratory Animal, College of Medicine, The Catholic University of Korea (approval number; CUMC-2009-0050-03).

### Cell culture and transfection

THLE-2 and THLE-3 (ATCC, Bethesda, MD, USA), which were derived from human primary normal liver cells, were cultured in Bronchial Epithelium Basal Medium (BEBM) (Lonza, Walkersville, MD, USA) supplemented with 10% fetal bovine serum (FBS) (Invitrogen, Carlsbad, CA, USA), 5 ng/mL epidermal growth factor (EGF) and 70 ng/mL phosphoethanolamine. Another normal liver hepatocyte, MIHA (kindly provided by Dr. Jayanta Roy-Chowdhury) was maintained in DMEM supplemented with 10% FBS, penicillin (100 U/mL) and streptomycin (100 µg/mL). Ten human HCC-derived cell lines; HepG2, Hep3B, PLC/PRF/5, SNU182, SNU354, SNU368, SNU387, SNU423, SNU449 and SNU475 (Korean Cell Line Bank [KCLB], Seoul, Korea) were maintained in RPMI-1640 supplemented with 10% FBS. The detailed information of these cell lines were listed in Table S1. Both HDAC1-specific and scrambled siRNA (negative control) were purchased from Genolution (Genolution Pharmaceuticals, Inc, Seoul, Korea) (Table S2). LC3B specific and negative control siRNA were synthesized (BIONEER Corp., Daejeon, Korea) (Table S2). Cells were transfected with siRNA using Lipofectamine™ 2000 (Invitrogen, San Diego, CA, USA) in accordance with the manufacturer's instructions.

### Plasmid preparation

The minimal luciferase reporter plasmid is Sp1-luc [36]. pWWP was generated by subcloning the 2.4 kbp human wild-type p21^WAF1/Cip1^ promoter into pGL3-Basic reporter vector, and three deletion-formed plasmids (pWPdel-BstXI, pWP101 and pWPdel-SmaI) and two mutant plasmids which have mutation on specific Sp1 binding site (pWPmt-Sp1-3 and pWPmt-Sp1-5,6) [36]. The HDAC1 shRNA and scrambled shRNA template oligos were synthesized and cloned into pSilencer 3.1-H1 neo plasmid (Ambion, Austin, TX, USA). Two pairs of oligonucleotide sequences for the templatd of shRNA vectors were listed in Table S3.

### MTT assay

3-(4, 5-dimethylthiazol-2-yl)-2, 5-diphenyltetrazolium bromide (MTT) assay was conducted to measure the number of viable cells. At the indicated time point, medium was replaced with the fresh medium containing 0.5 mg/mL of MTT (Sigma-Aldrich, St. Louis, MO, USA). After 4 h incubation at 37°C, the cellular formazan product was dissolved in dimethylsulfoxide (DMSO), and the absorbance was measured at a wavelength of 570 nm by using spectrophotometry (PerkinElmer Inc, Boston, MA, USA).

### Analysis of cell cycle distribution and apoptosis by flow cytometry

Hep3B cells were transfected with either HDAC1 siRNA (si-HDAC1) or scrambled siRNA (si-Scr) in 60-mm dishes. After 48 h incubation, transfected cells were collected and washed twice with PBS. Subsequently, cells were fixed in 70% ethanol overnight at −20°C. Fixed cells were then washed once in ice-cold PBS and stained with propidium iodide (PI) staining solution (50 µg/mL PI, 100 µg/mL RNase, 0.05% Triton X-100 in PBS). PI-stained cells were then analyzed for their DNA content by using a FACSCalibur flow cytometry with CellQuest™ software (BD Biosciences, San Jose, CA, USA). Annexin V-FITC kit (BD Biosciences) was used to measure the percentage of apoptosis. After 72 h post-transfection of HDAC1 siRNA, Hep3B cells were harvested and resuspended in 100 µL of binding buffer (1×10^6^ cells/mL) containing 5 µL of Annexin V-FITC and 5 µL of PI. After incubation away from light for 15 min at room temperature, the stained cells were analyzed by FACSCalibur and FlowJo software (Tree Star).

### qRT-PCR

Total RNA was isolated by using TRIzol (Invitrogen) as described by the manufacturer. cDNA was generated by Transcriptor First Strand cDNA Synthesis Kit (Roche Applied Science, Indianapolis, IN, USA). Relative levels of specific mRNA were determined with a SYBR Green chemistry system. All PCRs were performed with the iQ™5 Real-Time PCR Detection System (Bio-Rad Laboratories, Philadelphia, PA, US) according to the manufacturer's instruction. The GAPDH (glyceraldehyde-3-phosphate dehydrogenase) gene was used as a control for normalization. PCR primers used were listed in Table S4.

### Western blot analysis

Cultured cells were washed with ice-cold PBS and cell lysates were prepared in RIPA buffer (50 mM Tris-Cl pH 7.4, 150 mM NaCl, 0.25% sodium deoxycholate, 1% NP-40, 100 µg/mL PMSF, 1×cØmplete Protease Inhibitor Cocktail (Roche Applied Science, Indianapolis, IN, USA)). Cell lysates were passed through 1 mL needle syringe to facilitate the disruption of the cell membranes and centrifuged at 14,000 rpm for 15 min at 4°C, and supernatants were collected. Protein concentrations of cell lysates were determined by Pierce® BCA Protein Assay Kit (Pierce, Rockford, IL, USA). Total protein was loaded onto an SDS-PAGE gel and transferred onto polyvinylidene difluoride (PVDF) membranes (Millipore). Membranes were blocked for 1 h at room temperature in 5% skim milk, washed with TBST (150 mM NaCl, 10 mM Tris pH 7.4, 0.1% Tween-20), and incubated with the indicated antibodies (Table S5). Membranes were washed three times in TBST and HRP-conjugated secondary antibody was added at 1∶5000 in 5% skim milk for 1 h at room temperature. Membranes were washed and processed with ECL Plus Western Blotting detection kit (Amersham Biosciences, Buckinghamshire, UK) and the signal was detected using an LAS-4000 image analyzer (Fuji Photo Film Co., Tokyo, Japan). Protein level was quantified by ImagJ solftware.

### ChIP-qPCR analysis

Cells were grown overnight in 100 mm dishes to 70–90% confluence, then cross-linked with formaldehyde, harvested, and chromatin immunoprecipitations were performed following the protocol for ChIP-qPCR analysis as described in our previous report [22]. For each IP, diluted chromatin was incubated with antibodies specific for HDAC1, Ac-histone H3 and Ac-histone H4 (Table S5) or normal rabbit IgG at 4°C for 2 h or overnight. The purified DNA was used as template for 60 cycles of PCR amplification using designated primers (Table S4).

### Luciferase reporter assay

The p21 promoter plasmids were transiently co-transfected into Hep3B cells with HDAC1 siRNA or scrambled siRNA. Luciferase activity was measured in a luminometer (Perkin Elmer Inc.) after 48 h post-transfection with Luciferase Reporter Assay System (Promega, Madison, WI, USA). The luciferase activities were normalized with the amount of protein in cell lysates.

### Transmission electron microscopy analysis

The Hep3B cells were grown on 6-well tissue culture plates and transfected with either HDAC1 siRNA or scrambled siRNA. Seventy-two hours after transfection, cells were fixed with 2% glutaraldehyde in 0.1 M cacodylate with 3 mM CaCl_2_ at 4°C overnight, and then fixed with 1% OsO_4_ for 60 min. Samples were dehydrated through an ethanol series and transferred into Epon resin. Ultrathin sections were cut with a diamond knife on a Reichert ultra microtome; the sections were mounted on Formvar single-hole grids or 150-mesh grids, stained with uranyl acetate and lead citrate, and then examined at 80 kV under electron microscope (JEOL JEM 1010).

### Immunofluorescence

Cells were seeded on circular microscope cover slips (Fisher) precoated with fibronectin (final concentration of 10 µg/mL) and UV light sterilized. Slides were placed into 6-well tissue culture plates (Falcon). After transfection of either HDAC1 or scrambled siRNA for 72 h, cells were washed with PBS for 3 times and fixed in 4% paraformaldehyde solution for 10 min at room temperature. After rinsing with PBS, the cells were permeabilized with 0.2% Triton X-100 for 10 min. Following another rinse with PBS, cells were blocked for 1 h at room temperature with 4% BSA-PBS solution. The LC3B antibody (concentration 1∶100) was diluted in blocking buffer and incubated at 4°C for overnight. The cover slips were washed with PBS and incubated with Alexa Fluor® 488-conjugated anti-rabbit IgG (Invitrogen) (concentration 1∶1000) for 1.5 h at room temperature. The cells were washed with PBS and counterstained with Hoechst (Invitrogen) diluted at 1∶10000. Cover slips were mounted onto the glass slides using Gel/Mount (Biomeda, Foster City, CA). All images were obtained using Zeiss Axio Imager M1 microscope (Carl Zeiss MicroImaging GmbH, Oberkochen, Germany).

### Establishment of stable cell lines

Hep3B cells were transfected with either HDAC1 or scrambled shRNA vector. Twenty four hours later, transfected cells were subcultured at 1∶10 dilution to new culture dishes. The culture medium containing 0.5 mg/mL geneticin (Invitrogen) was changed every 3 days for 2 to 3 weeks until colonies of resistant cells formed.

### *In vivo* tumor xenograft study

Six week-old athymic nude mice were housed in filter-topped cages and received food and water ad labium. Tumors were generated by subcutaneous injection (s.c.) into the right lower flank with 5×10^6^ of Hep3B cells suspended in 100 µL PBS and mixed with 20% matrigel. After five to seven weeks from the cell inoculation, palpable tumours were established. Tumor dimensions were measured two times per week with a caliper. Tumor volumes were estimated as follows: V (mm^3^) = (length×width^2^)/2. Tumor weights were recorded at the time of sacrifice in order to evaluate HDAC1 knockdown response.

### Colony forming assay

The established stable Hep3B cells were cultured in a 6-well tissue culture plate at 1500 cells/well. Cells were grown for 10–14 days with geneticin at 0.5 mg/mL. Colonies were fixed by 4% paraformaldehyde for 30 min and stained with 0.5 mL of 0.005% Crystal Violet to enable enumeration of colonies. The colonies were counted as reported before [37].

### Statistical analysis

Unless otherwise stated, each experiment was performed at least three times and all the values were expressed as mean ± SD. The differences between the groups were compared using Student's *t*-test. *p*<0.05 was considered statistically significant.

## Supporting Information

Table S1 **Information of human liver cell lines.**
(XLS)

Table S2 **siRNA sequences used in this study.**
(XLS)

Table S3 **Oligonucleotide sequences for shRNA plasmid construction.**
(XLS)

Table S4 **Primer information in this study.**
(XLS)

Table S5 **Antibodies used in the present study.**
(XLS)
